# Concurrent Implicit Adaptation to Multiple Opposite Perturbations

**DOI:** 10.1523/ENEURO.0066-23.2023

**Published:** 2023-08-04

**Authors:** Pierre-Michel Bernier, Alice Puygrenier, Frederic R. Danion

**Affiliations:** 1 Département de Kinanthropologie, Université de Sherbrooke, Sherbrooke, Québec, J1K 2R1, Canada; 2 Centre National de la Recherche Scientifique, Université de Poitiers, Université de Tours, Centre de Recherches sur la Cognition et l'Apprentissage, Unité Mixte de Recherche 7295, 86073 Poitiers Cedex 9, France

**Keywords:** interference, motor memories, reaching, visuomotor adaptation, dual adaptation

## Abstract

Simultaneous adaptation to opposite visuomotor perturbations is known to be difficult. It has been shown to be possible only in situations where the two tasks are associated with different contexts, being either a different colored background, a different area of workspace, or a different follow-through movement. However, many of these elements evoke explicit mechanisms that could contribute to storing separate (modular) memories. It remains to be shown whether simultaneous adaptation to multiple perturbations is possible when they are introduced in a fully implicit manner. Here, we sought to test this possibility using a visuomotor perturbation small enough to eliminate explicit awareness. Participants (*N* = 25) performed center-out reaching movements with a joystick to five targets located 72° apart. Depending on the target, visual feedback of cursor position was either veridical (one target) or could be rotated by +5 or −5° (two targets each). After 300 trials of adaptation (60 to each target), results revealed that participants were able to fully compensate for each of the imposed rotations. Moreover, when veridical visual feedback was restored, participants exhibited after-effects that were consistent with the rotations applied at each target. Questionnaires collected immediately after the experiment confirmed that none of the participants were aware of the perturbations. These results speak for the existence of implicit processes that can smoothly handle small and opposite visual perturbations when these are associated with distinct target locations.

## Significance Statement

Simultaneous adaptation of reaching movements to opposite visuomotor perturbations has been shown to be possible mainly in situations where the two tasks are associated with different contexts. However, the relative contribution of explicit and implicit mechanisms has remained unclear. Here, by introducing visuomotor rotations small enough to rule out the implication of explicit mechanisms, we show that implicit processes alone are sufficient to handle multiple (even opposite) perturbations.

## Introduction

Many studies have demonstrated that humans can adapt their visuomotor map to biased visual feedback of their hand when performing spatially directed reaching movements (Cunningham, 1989; Krakauer et al., 2000; Abeele and Bock, 2001; Sainburg and Wang, 2002; Tong and Flanagan, 2003; Krakauer, 2009; Henriques and Cressman, 2012). In most cases, a single perturbation is applied to the entire workspace, thus requiring a simple transformation of the visuomotor map. The situation becomes more challenging when a perturbation experienced at one location is different (possibly opposite) from that at another location (Ghahramani et al., 1996; Woolley et al., 2007; Thomas and Bock, 2012; Schween et al., 2018; Tsay et al., 2023), as this imposes specific adjustments of hand movements depending on the target to be reached. Still, several studies have shown that participants can regain accuracy in such context.

In a seminal study by Ghahramani et al. (1996), the authors introduced perturbations that consisted in shifting hand visual feedback in opposite directions in two parts of the workspace. Namely, participants had to point 10 cm toward their body to achieve a target in the left part of the workspace, and 10 cm away from their body to achieve a target in the right part of the workspace. Results revealed that participants could simultaneously adapt their movements to those opposite perturbations. The pattern of generalization further showed that changes were largest near the practiced targets and decreased away from them, demonstrating the local nature of the changes in the visuomotor map. Later, Woolley et al. (2007) showed that dual adaptation to opposite visuomotor rotations is also possible when they are associated with different regions of the workspace. In that study, the authors employed a center-out reaching task in which two diametrically opposed quadrants were associated with 30° visuomotor rotations of opposite sign. Similarly, Thomas and Bock (2012) demonstrated that participants could simultaneously adapt to four perturbations (−60°, −30°, +30°, +60°) when cued by workspace location (distinct target) and by the arm used. More recently, using one arm only, it was shown that participants can adapt to opposite visuomotor rotations (+45°, and −45°) when each one is associated with a different region of the workspace (Schween et al., 2018). Even more recently, it was shown to be possible to simultaneously adapt to three perturbations of the same sign but different sizes (+30°, +45°, and +60°) when reaching to targets spread over three regions 120° apart (Tsay et al., 2023).

In parallel to these studies, it has been well documented that visuomotor adaptation is driven by both explicit processes (e.g., strategic re-aiming) and implicit processes (Hwang et al., 2006; Mazzoni and Krakauer, 2006; Hegele and Heuer, 2010; Taylor et al., 2014; Hutter and Taylor, 2018). Given the large magnitude and abrupt onset of the perturbations employed in most of the studies cited above (10 cm shift; >30° rotations), as well as the limited number of targets which facilitates deployment of strategies, it has been hard to disentangle the extent to which dual adaptation is attributable to the explicit awareness of a change in context, as compared with purely implicit processes (Hegele and Heuer, 2010).

To address this issue, we designed a task in which participants performed center-out reaching movements toward five peripheral targets each separated by 72°. The use of five targets spread around 360° was meant to minimize the use of explicit strategies, as it becomes more complex to adopt a cognitive strategy when the number of targets increases (Forano et al., 2021). A key manipulation as compared with previous work was to use very small cursor rotations, of +5°, 0°, or −5° depending on the target, which we reasoned (and later confirmed) would eliminate explicit awareness. Together, the finer fragmentation of the workspace and smaller perturbations provided a strong test for dual implicit adaptation. If concurrent implicit adaptation to multiple perturbations occurs, then we predicted that with training, directional errors would decrease for all rotated targets, and be followed by after-effects opposite to the induced perturbations following their removal. We further predicted that there would be no directional change whatsoever for the nonrotated target throughout training. As will be shown, these observations were confirmed with participants smoothly and implicitly adapting their reaches to the local constraints associated with each target. These results demonstrate that implicit processes are sufficient to handle multiple (even opposite) perturbations.

## Materials and Methods

### Participants

Twenty-five healthy right-handed volunteers were recruited (19.9 ± 1.5 years, 10 females). Handedness of participants was verified using the Oldfield Handedness Inventory (Oldfield, 1971) with a mean laterality index of 86.8 ± 7.8%. All participants gave written consent before participation. The experimental paradigm was approved by the local ethics committee (IRB00012476-2022-29-11-210) and complied with the Declaration of Helsinki.

### Data acquisition

**Figure 1.**
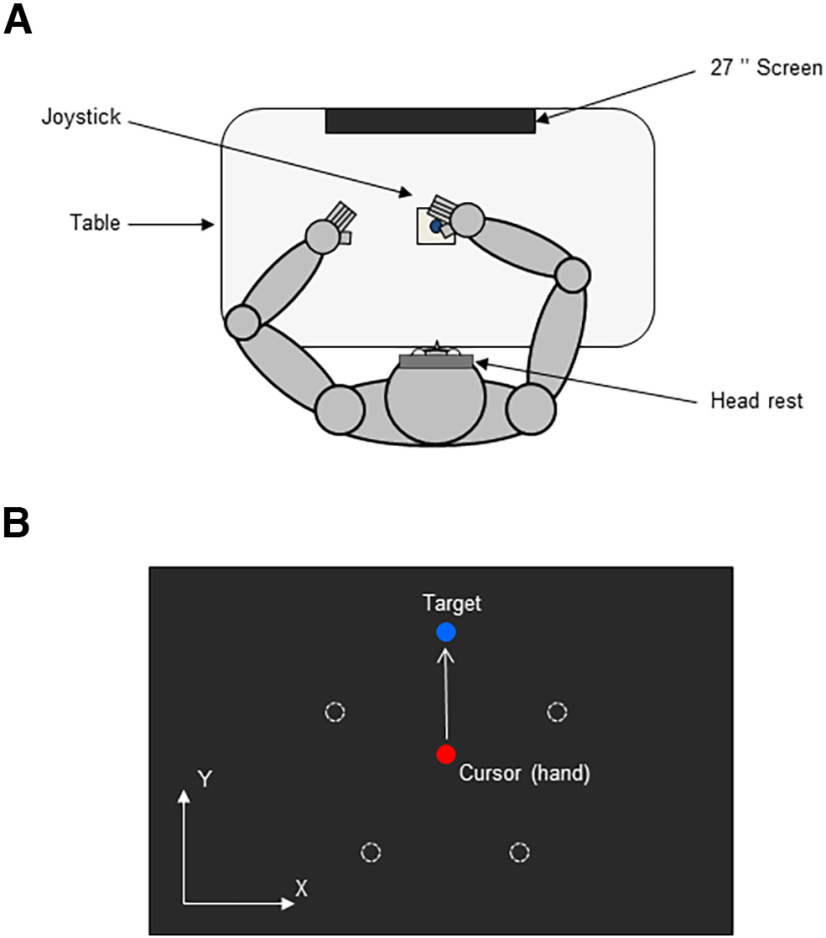
Schematic drawing of the experimental setup. ***A***, Top view of the participant sitting in the experimental setup. ***B***, Schematic view of the screen during the reaching task (for more details, see Materials and Methods). Hollow white targets are displayed for illustration purposes (i.e., not visible during the actual task).

Figure 1*A* shows the experimental setup. Participants were seated comfortably in a dark room facing a screen (ACER predator, 1920 × 1080, 27 inches, 240 Hz) positioned on the frontal plane 57 cm away from the participant's eye. Participants’ head movements were restrained by a chin rest and a padded forehead rest so that the eyes in primary position were directed toward the center of the screen. In order to block vision of their hands, a mask was positioned under the participants’ chin. They were required to hold with the right hand a joystick (Serie 812, Megatron, France, with ±25° of rotation along *x-y*-axes) positioned horizontally on a table in front of the participant, in line with central sagittal plane. The joystick was spring loaded with a restoring force that was low but sufficient to bring back the joystick at its central position when the participant releases it. Both right and left forearms were resting on the table. The output of the joystick was recorded at 1000 Hz. Eye movements were not recorded; moreover, no explicit instruction was given regarding the point of gaze.

### Experimental design

Participants had to perform reaching movements with their right hand. They were instructed to perform fast and accurate center-out pointing movements (see Fig. 1*B*). At the beginning of each trial the participant was instructed to release the joystick to allow the red cursor to move at the center of the screen. Then after a fixed delay (2 s), a blue target appeared at the periphery. As soon as this peripheral target was visible, participants were required to make a fast and accurate pointing movement toward it. The target appeared at one of five possible locations (72° apart) that were spread 10 cm around the start position. Participants were encouraged to produce a quick uncorrected movement toward the target and were specifically instructed to refrain the release of final adjustments. After hand movement stops, participants had to release the joystick so that a new trial could start (3 s after the target was presented). The position of the target was randomized, but after every block of five trials, each of the five possible targets was presented once. We also ensured that a target was never presented twice in a row. The order of targets was randomized across blocks. The same target order was kept for all the participants, allowing greater statistical power when examining possible carry-over effects between targets.

**Figure 2.**
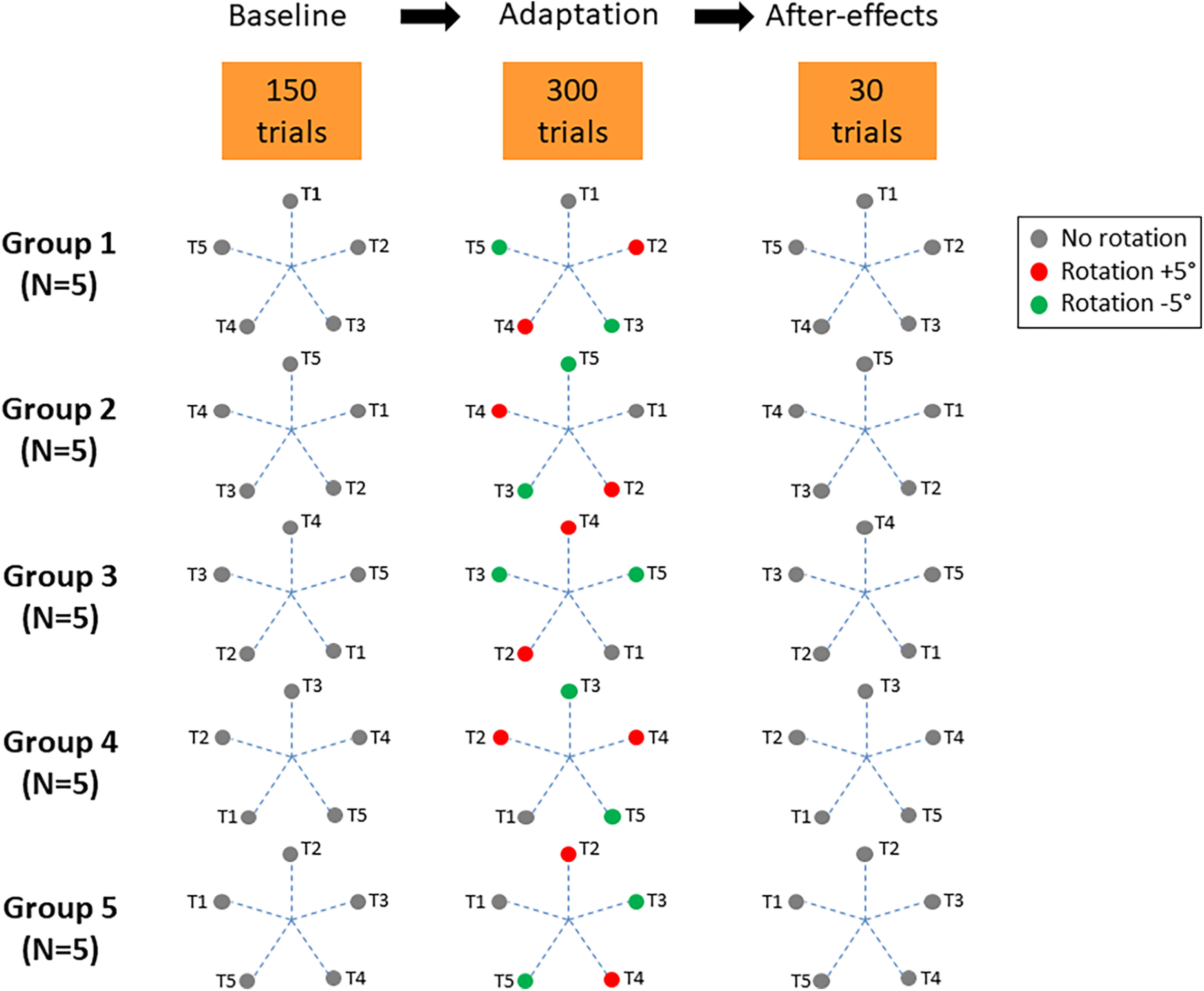
Experimental design for each group of participants. During the adaptation phase, depending on the target to be reached, visual feedback of cursor was either biased by +5° (red circle), by −5° (green circles), or remained veridical (gray circle). See Materials and Methods for further information.

**Figure 3.**
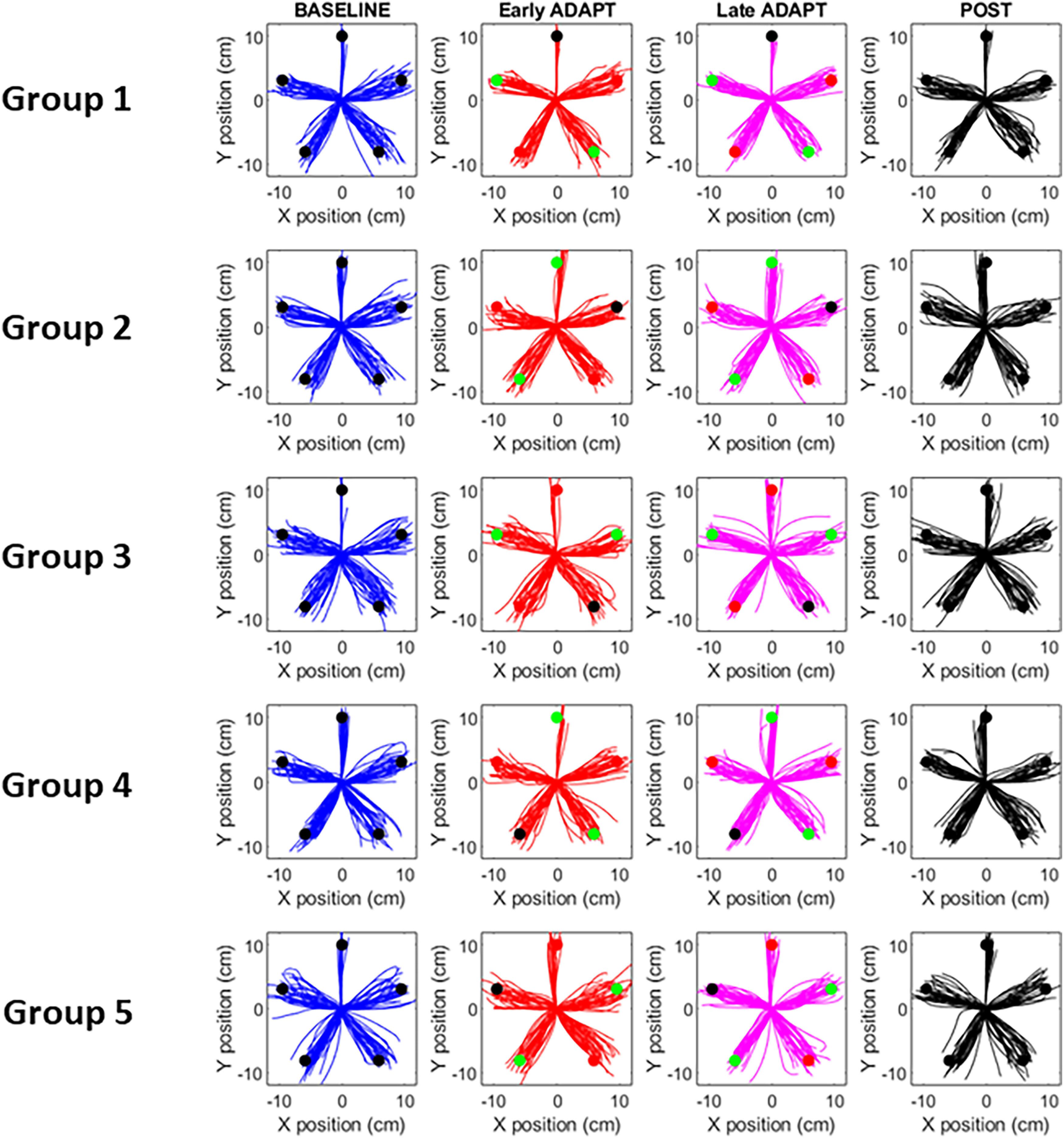
Reaching movement trajectories at various stages of the adaptation protocol for each of the five groups of participants. For each phase, and each target, we present six trials per participant. Circles indicate the position of the target, with color indicating the nature of the perturbation (red = +5°, green = −5°, black = 0°).

Under baseline condition, the relation between the joystick motion and its visual consequences on the screen was intuitive, mimicking the behavior of a computer mouse, with forward/backward joystick movements eliciting up/down cursor movements on the screen, and left/right joystick movements eliciting left/right cursor movements. Under adaptive condition, depending on the target, cursor visual feedback could be distorted by a ±5° rotation (a positive value indicating a counter-clockwise rotation). Specifically, Targets 2 and 4 were submitted to a +5° rotation, whereas Targets 3 and 5 were submitted to a −5° rotation (see Fig. 2). In contrast Target 1 was not submitted to rotation. Subjects were not informed about the possible rotations provided during the exposure session. As an attempt to neutralize possible effects inherent to hand and joystick anisotropy when reaching toward the different target locations (i.e., see the lower directional variability when reaching the top target in Fig. 3), participants were split into five groups (each *N* = 5, three males and 2 females). Relative position (and possible bias) of the five targets was maintained, but this pattern was rotated by steps of 72° across groups so that each target occupied the five possible positions (see Fig. 3).

The experimental session consisted of three phases (see Fig. 2). During the initial phase (Baseline, no rotation), participants performed one block of 150 trials under the regular mapping. Subsequently, during the adaptation phase, participants performed one block of 300 trials. During the final phase (post session), the initial mapping was restored allowing to test for after-effects with 30 trials. Altogether, each participant performed a total of 480 reaching trials.

### Data analysis

To assess reaching performance in each trial, we measured the directional error, the spatial error at the end of the movement, as well as reaction time and movement time. These variables were extracted as follows. Beginning and end of movement were estimated based on cursor tangential velocity, using a threshold at 8% of maximal peak velocity (Mutha et al., 2013). Determining those instants allowed to compute reaction time and movement duration. Final position error was evaluated through the Euclidian cursor-target distance at the end of movement (Wang and Sainburg, 2004). Cursor-path directional error was evaluated at maximal tangential velocity (Wang and Sainburg, 2009). It was calculated as the angle between the vector going from initial starting cursor position to cursor position at maximal velocity, and the vector going from the start position to target location. Trials were excluded whenever one of the following criteria was not met: reaction time outside of the 150- to 500-ms interval, movement time >400 ms, directional error >45°, and when spatial error was >5 cm. This procedure led to the removal of 373 trials out of the 12,000 trials collected over the 25 participants (3.1%).

Repeated measures ANOVAs were used as the main tool for statistical analyses. To assess the presence of adaptation for each target, we compared PRE (last six baseline trials), EARLY adaptation (six first adaption trials), LATE adaptation (last six adaptation trials), and POST phases (last six trials of post session). Newman–Keuls corrections were used for *post hoc t* tests to correct for multiple comparisons. A conventional 0.05 significance threshold was used for all analyses.

## Results

### General overview

Figure 3 plots all reaching trials performed by each group during baseline, early exposure, late exposure, and return to baseline (post session). As can be seen reaching performance was substantially altered when the rotation was first introduced (see red and green targets during early exposure). However, over the course of training, the detrimental effect of the rotation was strongly reduced (see red and green targets during late exposure). Subsequently when the rotation was removed (post session), after-effects were observed in the opposite direction. In the next sections, we analyze in more details these qualitative observations.

**Figure 4.**
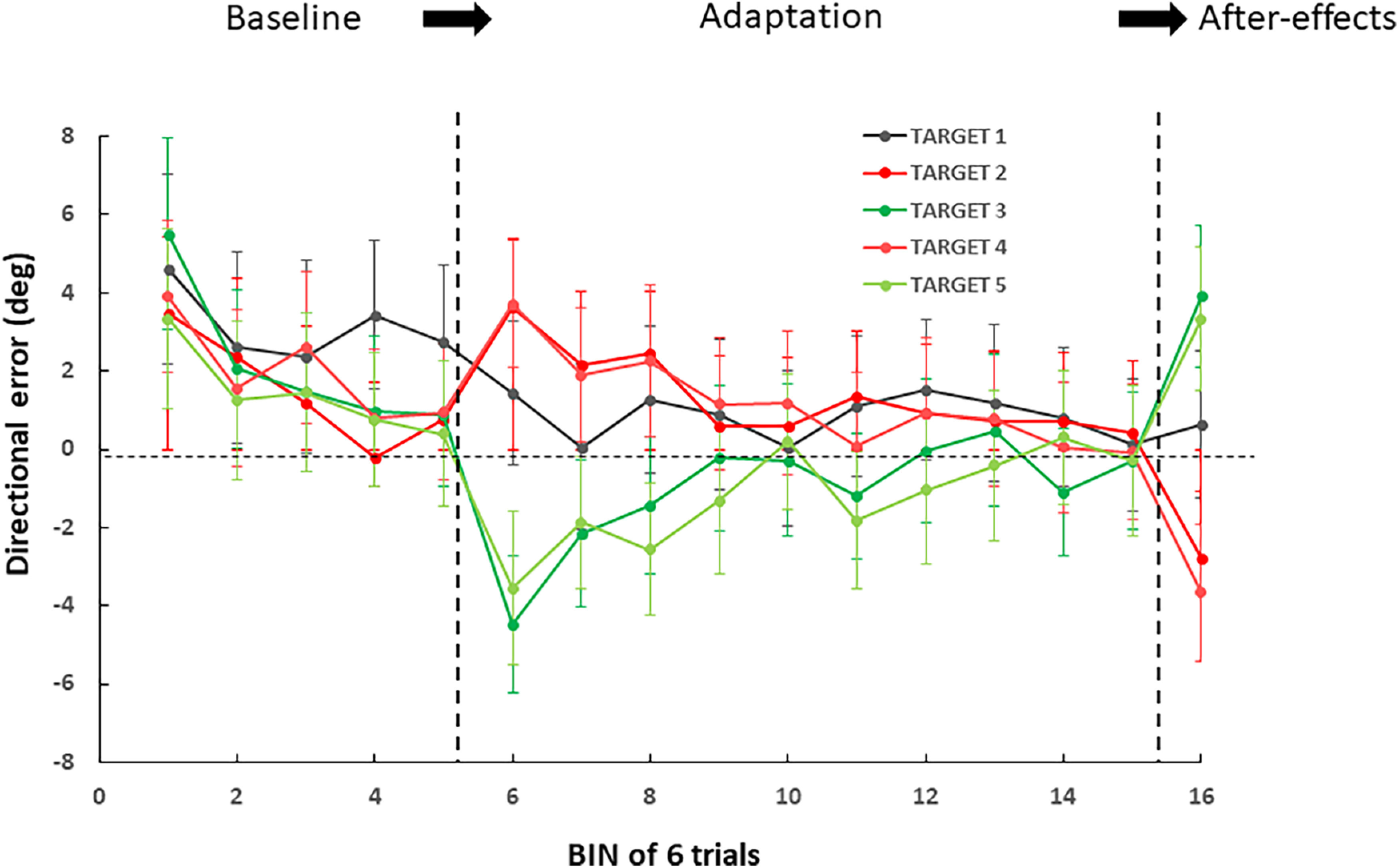
Time course of directional error as a function of bin (mean of 6 trials) and target. Errors bars correspond to SEM.

In Figure 4 we present the timeline of directional error over the whole experiment, for each target separately. As can be seen, for Targets 2–4, directional error increased substantially at the initiation of the adaptation phase. However directional error decreased throughout practice, up to a point where it was indistinguishable from the baseline phase. In the postadaptation phase, robust after-effects were observed in the opposite direction, suggestive of implicit visuomotor adaptation. In contrast, no similar trend was observed for Target 1, for which visual feedback was not perturbed. Below we examine, separately for each target, adaptation and after-effects in more detail.

### Target 1 (no rotation)

We propose first to examine reaching movements performed toward Target 1, for which visual feedback was not altered during the adaptation phase, and thus did not require any corrective actions. One-way ANOVA of directional error revealed no main effect of PHASE (BASE = 2.8°, EARLY = 1.3°, LATE = 0°, POST = 0.7°; *F*_(3,72)_ = 2.22; *p* = 0.09). This analysis is consistent with the view that participants did not adapt hand movements when reaching Target 1, and that there was virtually no detrimental effect from adjacent targets that were rotated.

### Targets 2 and 4 (+5° rotation)

Both Targets 2 and four were associated with a +5° rotation during the adaptation phase, thereby requiring corrective actions to maintain reach accuracy. Regarding Target 2, one-way ANOVA revealed a main effect of PHASE (BASE = 1.0°, EARLY = 3.7°, LATE = 0.6°, POST = −2.6°; *F*_(3,72)_ = 8.49; *p* < 0.001). *Post hoc* analyses revealed that all phases were different from each other (*p* < 0.05) except BASE and LATE (*p* = 0.72). Very similar results were obtained for Target 4. Indeed the ANOVA also revealed a main effect of PHASE (BASE = 1.0°, EARLY = 3.8°, LATE = 0°, POST = −3.8°; *F*_(3,72)_ = 18.81; *p* < 0.001), with *post hoc* analyses indicating that all phases were different from each other (*p* < 0.01) except BASE and LATE (*p* = 0.31). Overall, despite the small magnitude of the visual perturbation, these analyses provide evidence of adaptive mechanisms aiming to reduce the early increase in directional error. The fact that we observed after-effects in the opposite direction is consistent with the view that implicit visuomotor adaptation was at play in that reduction of directional error. The fact that directional error at the end of the adaptation phase was similar to that during baseline suggests that compensation of the +5° rotation was complete. To ensure the presence of adaptation, we also compared hand heading angle during BASE and LATE. For Target 2, the ANOVA revealed a shift of −5.4° in heading angle consistent with the +5° rotation (*F*_(1,24)_ = 12.68, *p* < 0.01). A similar shift (−6.0°) was observed for Target 4 (*F*_(1,24)_ = 40.61, *p* < 0.001).

### Target 3 and 5 (−5° rotation)

Targets 3 and 5 were both associated with a −5° rotation during the adaptation phase. Regarding Target 3, one-way ANOVA revealed a main effect of PHASE (BASE = 0.9°, EARLY = −4.4°, LATE = −0.5°, POST = 3.7°; *F*_(3,72)_ = 19.46; *p* < 0.001). *Post hoc* analyses revealed that all phases were different from each other (*p* < 0.05) except BASE and LATE (*p* = 0.20). Very similar results were obtained for Target 5 for which the ANOVA also revealed a main effect of PHASE (BASE = 0.4°, EARLY=−3.5°, LATE=−0.2°, POST = 3.4°; *F*_(3,72)_ = 11.05; *p* < 0.001) with *post hoc* analyses indicating significant differences between all phases (*p* < 0.05) except between BASE and LATE (*p* = 0.60) thereby supporting the view that compensation of the rotation was complete. Evidence for a change in hand heading angle between BASE and LATE was also obtained for both Target 3 (+3.6°; *F*_(1,24)_ = 7.91, *p* < 0.01) and Target 5 (+4.4°; *F*_(1,24)_ = 10.50, *p* < 0.01) in agreement with the −5° rotation. Overall, these results qualitatively mirror those previously reported for Targets 2 and 4.

### Questionnaires

To investigate the implicit/explicit nature of visual perturbations, participants were interrogated at the end of the experiment. A questionnaire was adapted from a previous study investigating explicit awareness during visuomotor adaptation (Benson et al., 2011). The first question was “Did you notice an increase in task difficulty at any given time?,” nine participants out of 25 answered yes. Among those participants, three of them stated that task difficulty increased during the middle of the experiment, two during the last part, one at the beginning, and three were unable to be specific. The second question was “Did you notice that the cursor was no longer moving where you intended?,” three participants out of 25 answered yes. When being asked “Any idea why?” most participants referred to a loss of concentration and fatigue. Two participants evoked a possible change in the gain of visual feedback (they felt they overshot), but none of them evoked a directional bias, let alone a target-specific bias. Altogether, the results provided by our questionnaire strongly suggest an absence of explicit awareness of the perturbation, and rather point to a predominant contribution of implicit adaptative mechanisms.

### Additional analyses

**Figure 5.**
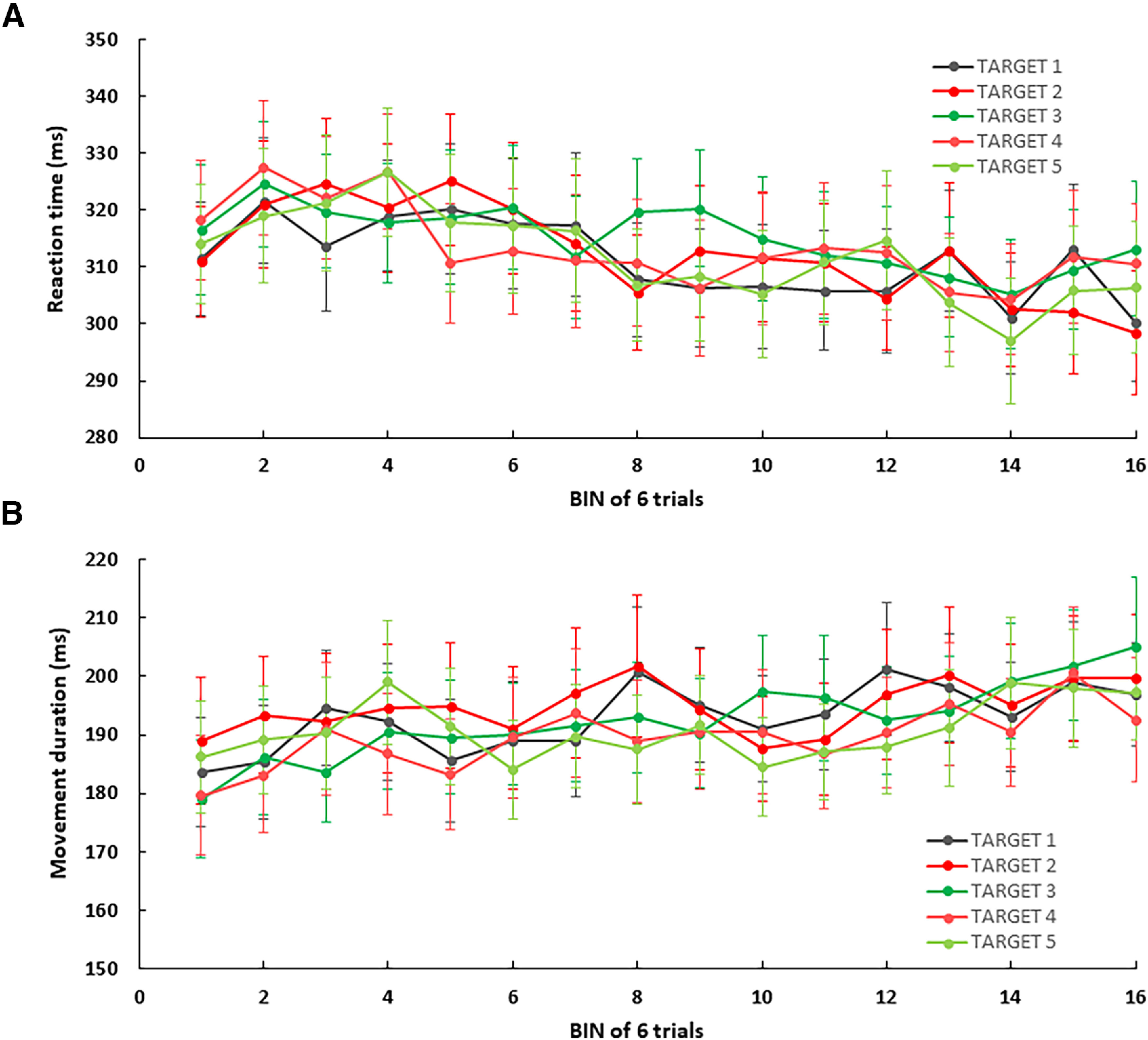
Time course of reaction time (***A***) and movement duration (***B***) as a function of bin (mean of 6 trials) and target. Errors bars correspond to SEM.

The possibility that participants implemented an explicit strategy (i.e., re-aiming), which requires time (Haith et al., 2015; McDougle and Taylor, 2019; Langsdorf et al., 2021), was further investigated by comparing their mean reaction times over the several phases of the experiment (see Fig. 5*A*). The analysis was performed by pooling reaction times over the four rotated targets (T2 to T5) as they exhibited similar overall values (*F*_(3,72)_ = 0.568, *p* = 0.638; T2 = 312, T3 = 317, T4 = 311, and T5 = 312 ms). One-way ANOVA revealed a main effect of PHASE *F*_(3,72)_ = 3.145; *p* = 0.03), indicating a tendency for faster reaction times as the protocol unfolded (see Fig. 5*A*). However, all *post hoc* comparisons failed to reach significance (closest being EARLY vs POST, *p* = 0.054). Importantly there was no difference between BASE and EARLY (318.2 vs 317.6 ms; *p* = 0.91). Moreover one-way ANOVA comparing reaction times across the five targets during the EARLY phase showed no significant difference between the rotated targets (T2 to T5) and the nonrotated target (T1) and (*F*_(4,96)_ = 0.56, *p* = 0.69; T1 = 321 ms, T2 = 320 ms, T3 = 321 ms, T4 = 312 ms, T5 = 318 ms). Given that re-aiming is typically associated with a lengthening in reaction time ranging from +200 to +400 ms (Saijo and Gomi, 2010; Fernandez-Ruiz et al., 2011; Rand and Rentsch, 2016), the lack of increase in reaction time observed in our study indirectly rules out the use of an explicit strategy (i.e., re-aiming). Still, if the increase in reaction time induced by strategic re-aiming scales with the magnitude of the rotation, it could be argued that any potential difference was harder to detect in our protocol compared with previous studies that employed larger perturbations (Saijo and Gomi, 2010; Fernandez-Ruiz et al., 2011; Rand and Rentsch, 2016).

When repeating the same analysis for movement duration (again pooled across Targets 2–5; see Fig. 5*B*), one-way ANOVA also revealed a main effect of PHASE (BASE = 190 ms, EARLY = 189 ms, LATE = 202 ms, POST = 199 ms; *F*_(3,72)_ = 2.79; *p* = 0.04), but none of the *post hoc* analyses reached significance (*p* > 0.067). Altogether these results speak for the consistency of reaching movements over the entire protocol, both at the planning level (by means of reaction times) and at the execution level (by means of movement duration).

Assuming that visuomotor adaptation is local, namely target-specific, we reasoned that adaptation to a given target should remain largely independent from the perturbation experienced at nearby targets. This means that adaptation at T2 and T4 (two targets exposed to a +5° rotation) should be identical despite the fact that each of these targets was surrounded by targets associated with different perturbations. Indeed, T2 was surrounded by T1 (no perturbation) and T3 (−5°), whereas T4 was surrounded by T3 and T5, two targets with −5° perturbations. To test whether the time course of adaptation was similar between T2 and T4, we performed a two-way ANOVA of directional error with TARGET (T2 vs T4) and PHASE. As expected, results showed a main effect of PHASE (*F*_(3,72)_ = 27.50, *p* < 0.001), but neither a main effect of TARGET (*F*_(1,24)_ = 0.106, *p* = 0.746), nor a PHASE by TARGET interaction (*F*_(3,72)_ = 0.23, *p* = 0.874). Very similar results were obtained when running the same analysis for T3 and T5. Indeed the two-way ANOVA showed a main effect of PHASE (*F*_(3,72)_ = 26.45, *p* < 0.001), but neither a main effect of TARGET (*F*_(1,24)_ = 0.003, *p* = 0.954), nor a PHASE by TARGET interaction (*F*_(3,72)_ = 0.306, *p* = 0.820). To summarize, the time course of adaptation at a given target was virtually unaffected by the perturbation experienced at the neighboring targets.

Along the same vein, we also evaluated the presence of carry-over effects during the adaptation phase as the perturbation experienced at one target may have influenced reaching performance on the next trial. Using the 300 adaptation trials, a first analysis consisted in contrasting directional error at T1 depending on whether the preceding reach was made toward T2, T3, T4, or T5. One-way ANOVA with TARGET (four levels) as within-subject factor showed no significant effect (*F*_(3,72)_ = 2.43, *p* = 0.07). Similar results were obtained when these analyses were repeated for the other (rotated) targets. Indeed no main effect of TARGET was observed for T2 (*F*_(3,72)_ = 0.764, *p* = 0.517), T3 (*F*_(3,72)_ = 0.100, *p* = 0.959), T4 (*F*_(3,72)_ = 2.215, *p* = 0.09), and T5 (*F*_(3,72)_ = 1.089, *p* = 0.359). Altogether, these analyses reveal no clear evidence of first-level history dependent effects, further supporting the notion that adaptation was local and target-specific.

Finally, the visual perturbation employed in this study was ±5°, and we sought to compare the magnitude of this shift to participants’ intrinsic reaching movement variability. To estimate individual variability, we computed for each participant and each target the SD of movement direction across the last 30 trials of baseline (i.e., last six trials made to each of the five targets). Averaged over the 25 participants, and the five targets, this procedure resulted in a mean variability of movement direction of 9.3 ± 1.4°, with individual values ranging from 5.7° up to 13.8°. A *t* test showed that such directional variability was significantly greater than 5° (*t*_(24)_ = 15.25, *p* < 0.001). Considering the magnitude of intrinsic variability, it makes it even more likely that introduction of the visuomotor rotation (±5°) remained unnoticed by participants.

## Discussion

The goal of this study was to investigate whether concurrent adaptation to multiple (opposite) perturbations is possible when they rely primarily on implicit adaptive mechanisms. Our results strongly support this possibility. Indeed, participants were able to restore accurate reaching direction according to the constraints of each target (+5° or −5°) and exhibited directionally-dependent after-effects when the perturbations were removed. Interestingly, the time course of adaptation at a given target was largely independent of the perturbation applied to adjacent targets, suggesting that adaptation was local. This is further supported by the fact that there was no change whatsoever for the target that was not perturbed (T1; 0°) and by the lack of carry-over effects from the perturbation experienced at a given target onto the next. Importantly, postexperiment questionnaires as well as analyses of reaction times and movement durations strongly suggest that participants were not explicitly aware of the perturbations and did not deploy strategic processes to counteract them. We propose now to discuss these observations, as well as the possible mechanisms allowing such flexibility in visuomotor adaptation.

Several observations support the view that visuomotor adaptation elicited by our protocol was largely implicit. First, our questionnaire revealed that participants were not aware of the perturbations, neither when they were introduced nor removed. Although postexperiment questionnaires have limitations (Taylor et al., 2014; Tsay et al., 2020), and perturbation awareness was not probed at the moment they were introduced (EARLY), our questions were still asked immediately after the post session, namely after an abrupt drop in performance (as evidenced by robust after-effects that persisted for over 30 trials), and yet none of the participants reported any awareness. The fact that baseline variability of reach direction (9.3°) was almost twice as large as the directional bias introduced by the rotations (5°) likely contributed to keeping perturbations unnoticed. Second, the analysis of reaction times and movement durations did not reveal any signs of longer planning or movement slowing that would be associated with strategic re-aiming (Haith et al., 2015; McDougle and Taylor, 2019; Langsdorf et al., 2021). It should also be reiterated that the use of multiple targets around 360° likely further minimized the adoption of a target-dependent cognitive strategy (Forano et al., 2021).

Altogether these observations speak for robust implicit adaptation, and also essentially rule out the possibility that explicit mechanisms contributed to the reduction in directional bias. Our findings are consistent with recent work looking at the generalization of aftereffects following visuomotor adaptation which has revealed that, when cued by workspace location, dual adaptation of an implicit visuomotor map is feasible (Schween et al., 2018). Similarly, although visuomotor and force field adaptation are thought to rely on partly distinct neural mechanisms (Krakauer et al., 1999; Donchin et al., 2012), our findings also resonate with recent work showing that dual force-field adaptation occurs primarily through implicit mechanisms when cued by workspace location (Forano et al., 2021). Still, even in those studies, large, detectable perturbations were used, hence the current work extends them in an important way by strongly eliminating any contribution from explicit awareness. The current findings also echo the observation that participants can effortlessly acquire multiple auditory-speech transformations simultaneously without explicit cues and while being unaware of the perturbations (Rochet-Capellan and Ostry, 2011).

Several observations also underpin the view that visuomotor adaptation elicited by our protocol was local. Indeed, despite the fact that two targets were deviated by +5° (T2 and T4), two others by −5° (T3 and T5), and one was not deviated (T1), the time course of directional error showed that adaptation was largely target specific (see Fig. 4). Moreover, reliable after-effects were observed in the expected direction (i.e., opposite to the rotation) for T2 to T5, but absent for T1. Finally, when comparing the time course of adaptation of T2 with that of T4, or T3 with that of T5, we found no significant differences, suggesting that neighboring targets had virtually no mutual influence. This view was further confirmed when examining our data for carry-over effects during adaptation. Indeed, we found no evidence that reaching performance at one target was influenced by the previously visited target (and thus perturbation). All these observations suggest that, for a given target, adaptive processes were exclusively driven by the error experienced at that target, and independent from those experienced at neighboring targets. In other words, visuomotor adaptation to the rotation was local.

The current findings support earlier observations showing that adaptation to multiple and opposite visuomotor rotations is possible when they are associated with different regions of workspace, or more specifically when the different targets are associated with different motor plans (Woolley et al., 2007; Thomas and Bock, 2012; Schween et al., 2018). However, they extend them in a critical way by using perturbations small enough to rule out the contribution of explicit mechanisms. Indeed, the demonstration of multiple adaptation in a purely implicit context places stronger constraints on the network's adaptive capabilities. A likely neural substrate that could mediate this effect is the primary motor cortex (M1). Tuning functions in M1 are thought to span roughly ∼60° around the preferred direction (Amirikian and Georgopoulos, 2000), implying that in the present context, movement planning and execution to each of the five targets (located 72° apart) was subtended by partially independent neuronal populations. It is well known that M1 undergoes structural changes specifically related to implicit visuomotor adaptation and that these changes subtend long-term storage of visuomotor memories (Paz et al., 2003; Hadipour-Niktarash et al., 2007; Hamel et al., 2017). Furthermore, the M1 neurons that undergo changes in activity following exposure to visuomotor rotations tend to be only those whose preferred direction corresponds to the adapted direction (Paz et al., 2003), supporting the specificity of the effect. Hence, it is likely that in the present context, movement-related activity at each target was subtended by separate neuronal populations whose changes in synaptic weights were updated according to each perturbation independently from each other, thereby leading to the multiple adaptation pattern observed here.

Traditionally, when investigating visuomotor adaptation to a rotated visual feedback, the magnitude of the rotation is >15°, whereas here we used a much smaller perturbation size. Still, the results were clear: not only did participants adjust their movements to the multiple rotations, but they fully compensated for the directional biases, allowing to restore baseline levels of performance. Moreover, when the perturbation was removed, reliable after-effects were observed in the opposite direction. Although few studies have investigated adaptation to such small perturbations introduced abruptly, protocols in which perturbations are introduced gradually as in a ramp (for instance 1° every 5 trials) have, by design, exposed participants to comparably small perturbations (Klassen et al., 2005; Hadipour-Niktarash et al., 2007; Werner et al., 2014). Yet, these protocols tend to assess the presence of after-effects only after reaching larger perturbations (>15°) and rarely after only 5°. A novel feature of the current study is to show that adaptation to an abruptly introduced 5° rotation is complete, as are the related after-effects.

As any experiment, the current one raises new questions. For one, the workspace used herein was segmented into five regions. While the use of five regions is more than previous studies on the matter (Woolley et al., 2007; Hegele and Heuer, 2010; Thomas and Bock, 2012; Schween et al., 2018; Tsay et al., 2023), whether similar results would hold with an even finer fragmentation of the workspace remains unknown. It has been advocated that visuomotor adaptation to a given target generalizes well to neighboring targets up to ∼30° apart (Krakauer et al., 2000; Brayanov et al., 2012; Fernandes et al., 2012, 2014; Day et al., 2016; McDougle et al., 2017; Zhou et al., 2017). However, heading direction can be biased for neighboring targets up to ∼45° apart (Krakauer et al., 2000; Fernandes et al., 2012, 2014). If correct, one may expect that dual adaptation becomes tricky, if not impossible, when increasing the number of targets, as interference between adjacent targets builds up. This scenario echoes with the observation that “visual targets nearest the workspace of the opposing visuomotor rotation exhibited the most interference” (Woolley et al., 2011). It also resonates with another study showing that dual force field adaptation is progressively impaired as training locations increase, a phenomenon well accounted by interference between the directional tuning functions recruited for each force field (Howard and Franklin, 2015). On the other hand, the fact that visuomotor adaptation generalizes ∼30° around the target has been put forward with perturbations much larger than the current one, and it is unclear whether generalization operates the same way when participants face smaller rotations as those used here.

A second issue relates to the potential computational cost associated with multiple adaptation as compared with adaptation to a single perturbation. While the present results do demonstrate that multiple implicit adaptation is possible, it is unknown whether adaptation would have been even faster had all five targets been rotated similarly (e.g., +5°). Under the assumption that adaptation is truly a local process (i.e., only driven by errors at that location), the overall rate of learning at a given target would be expected to be similar regardless of whether a single or multiple perturbations are used.

In conclusion, the current study provides further evidence that simultaneous adaptation of reaching movements to opposite visuomotor perturbations is possible. However, in contrast to previous studies in which the contribution of explicit mechanisms could not be ruled out, we show that dual adaptation remains possible even when this contribution is strongly minimized. Specifically, by using visuomotor rotations small enough to rule out the implication of explicit mechanisms, and by using a large set of targets, which makes it more difficult to adopt a cognitive strategy, we show that implicit processes can smoothly handle multiple (even opposite) visual perturbations when these are associated with distinct target locations/workspace regions. Moreover, in contrast to previous studies that employed larger rotations, we show that implicit compensation of a directional bias can be complete. Altogether, this study encourages further work using smaller visual perturbations, and greater fragmentation of the workspace, as this may offer new insights about visuomotor adaptation.

## Synthesis

Reviewing Editor: David Franklin, Technische Universitat Munchen Decisions are customarily a result of the Reviewing Editor and the peer reviewers coming together and discussing their recommendations until a consensus is reached. When revisions are invited, a fact-based synthesis statement explaining their decision and outlining what is needed to prepare a revision will be listed below. The following reviewer(s) agreed to reveal their identity: Jonathan Tsay, Ian Howard.

Overall, the reviewers were happy with the revisions to your manuscript.

Reviewer 1:

Thank you for addressing my comments. I have no further suggestions at this time.

Reviewer 2:

The authors have essentially addressed my concerns. The title has certainly now also improved. I think there are still aspects of the study which are limited in their novelty. However, overall, the work was well carried out and includes further references to previous work. The study did involve the use of small perturbations to minimize much as possible the use conscious explicit strategies. As such the work does contribute to the study to implicit and explicit learning.
